# The Jean Gutierrez spider mite collection

**DOI:** 10.3897/zookeys.489.9292

**Published:** 2015-03-23

**Authors:** Alain Migeon

**Affiliations:** 1 INRA, UMR 1062 CBGP, F-34988 Montferrier-sur-Lez, France

**Keywords:** Acari, Tetranychidae, World, Madagascar, Western Indian Ocean, New Caledonia, South Pacific, Papuasia

## Abstract

The family Tetranychidae (spider mites) currently comprises 1,275 species and represents one of the most important agricultural pest families among the Acari with approximately one hundred pest species, ten of which considered major pests. The dataset presented in this document includes all the identified spider mites composing the Jean Gutierrez Collection hosted at the CBGP (Montferrier-sur-Lez, France), gathered from 1963 to 1999 during his career at the Institut de Recherche pour le Développement (IRD). It consists of 5,262 specimens corresponding to 1,564 occurrences (combination species/host plant/date/location) of 175 species. Most specimens were collected in Madagascar and other islands of the Western Indian Ocean, New Caledonia and other islands of the South Pacific and Papuasia. The dataset constitutes today the most important one available on Tetranychidae worldwide.

## Data published through GBIF

http://www.gbif.org/dataset/ac60a288-fcc9-43fe-a7d4-e732b748a981

## Project details

**Project title:** Spider mites collection of Jean Gutierrez.

**Personnel:** Alain Migeon (data manager, data publisher, supervisor), Franck Dorkeld (computer specialist), Jonathan Bonfanti (data entry).

**Funding:** GBIF France and Institut National de la Recherche Agronomique (INRA).

**Design description:** This dataset was developed to increase the knowledge of an important agricultural pest family, the spider mites (Arthropoda, Acari, Tetranychidae). This family contains 1,275 species (Migeon and Dorkeld 2006–2013), among which one hundred can be considered as pests, ten of which major pests. The spider mite collection has been established by Jean Gutierrez, acarologist of the Institut de Recherche pour le Développement (IRD) from 1963 to 1999 and is presently hosted at CBGP (CBGP – INRA, Campus International de Baillarguet, 755 Avenue du Campus Agropolis, CS 30016, 34988 MONTFERRIER-sur-LEZ Cedex, France), an INRA and IRD laboratory in Montpellier. The collection contains 5,262 slides representing 1,564 occurrences (species/host plant/date/location). This collection represents a unique source of data for this family in Madagascar and New Caledonia and a major source for Pacific Islands and Mascarens Islands. The dataset should contribute to a much better understanding of this mite family in addition to the taxonomic database hosted by INRA (Migeon and Dorkeld 2006–2013).

## Taxonomic coverage

### General taxonomic coverage description

All the recorded specimens in the dataset were identified to species. The identification of spider mites to species often requires the examination of male genitalia and specimens identified to genus were generally single females and have been discarded. Unidentified specimens have also been discarded. The dataset contains 175 species, i.e. 14 % of the species known in this family. Jean Gutierrez described 50 species (Table 1). Types of 49 are deposited in his collection.

**Table 1.** Tetranychidae species described by Jean Gutierrez with respective title and source of the publication and present nomenclature (Blommers and Gutierrez 1975; Gutierrez 1966; 1967a; b; 1968a; b; 1969; 1970; 1972; 1977; 1978; 1982; Gutierrez and Bolland 1973; 1986; Gutierrez and Etienne 1981; Gutierrez and Helle 1971). The presence of the type specimen and the number of types and paratypes in the dataset is also indicated.

Original genus	Species	Author	Present combination	Publication title	Publication source	Type	Number of types and paratypes specimens
*Eonychus*	*grewiae*	Gutierrez, 1969	*Eonychus grewiae*	Tetranychidae nouveaux de Madagascar (Cinquième note)	Acarologia, 11: 43–64	yes	17
*Eotetranychus*	*befandrianae*	Gutierrez, 1967	*Eotetranychus befandrianae*	Huit nouvelles espèces du genre *Eotetranychus* Oudemans (Acariens: Tetranychidae) de Madagascar	Acarologia, 9: 370–394	yes	15
*Eotetranychus*	*borbonensis*	Gutierrez, 1968	*Eotetranychus borbonensis*	Note sur quelques acariens phytophages de l’Ile de la Réunion avec description d’une nouvelle espèce du genre *Eotetranychus* Oudemans (Tetranychidae)	Acarologia, 10: 444–446	yes	47
*Eotetranychus*	*botryanthae*	Gutierrez, 1970	*Eotetranychus botryanthae*	Tetranychidae nouveaux de Madagascar (Sixième note)	Acarologia, 12: 714–731	yes	23
*Eotetranychus*	*capricorni*	Gutierrez, 1967	*Eotetranychus capricorni*	Huit nouvelles espèces du genre *Eotetranychus* Oudemans (Acariens: Tetranychidae) de Madagascar	Acarologia, 9: 370–394	yes	8
*Eotetranychus*	*friedmanni*	Gutierrez, 1968	*Eotetranychus friedmanni*	Tetranychidae nouveaux de Madagascar (Quatrième note)	Acarologia, 10: 13–28	yes	105
*Eotetranychus*	*garnieri*	Gutierrez, 1978	*Eotetranychus garnieri*	Cinq nouvelles espèces de Tetranychidae (Acariens) de Nouvelle-Calédonie	Acarologia, 20: 351–364	yes	19
*Eotetranychus*	*grandis*	Gutierrez, 1969	*Eotetranychus grandis*	Tetranychidae nouveaux de Madagascar (Cinquième note)	Acarologia, 11: 43–64	yes	10
*Eotetranychus*	*greveanae*	Gutierrez, 1970	*Eotetranychus greveanae*	Tetranychidae nouveaux de Madagascar (Sixième note)	Acarologia, 12: 714–731	yes	10
*Eotetranychus*	*limoni*	Blommers & Gutierrez, 1975	*Eotetranychus limoni*	Les tétranyques vivant sur agrumes et avocatiers dans la région de Tamatave (Madagascar-est) et quelques-uns de leurs prédateurs	Fruits, 30: 191–200	yes	23
*Eotetranychus*	*paracybelus*	Gutierrez, 1967	*Eotetranychus paracybelus*	Huit nouvelles espèces du genre *Eotetranychus* Oudemans (Acariens: Tetranychidae) de Madagascar	Acarologia, 9: 370–394	yes	50
*Eotetranychus*	*pauliani*	Gutierrez, 1968	*Eotetranychus pauliani*	Tetranychidae nouveaux de Madagascar (Quatrième note)	Acarologia, 10: 13–28	yes	51
*Eotetranychus*	*rinoreae*	Gutierrez, 1970	*Eotetranychus rinoreae*	Tetranychidae nouveaux de Madagascar (Sixième note)	Acarologia, 12: 714–731	yes	30
*Eotetranychus*	*robini*	Gutierrez, 1978	*Eotetranychus robini*	Cinq nouvelles espèces de Tetranychidae (Acariens) de Nouvelle-Calédonie	Acarologia, 20: 351–364	yes	19
*Eotetranychus*	*roedereri*	Gutierrez, 1967	*Eotetranychus roedereri*	Huit nouvelles espèces du genre *Eotetranychus* Oudemans (Acariens: Tetranychidae) de Madagascar	Acarologia, 9: 370–394	yes	14
*Eotetranychus*	*sakalavensis*	Gutierrez, 1967	*Eotetranychus sakalavensis*	Huit nouvelles espèces du genre *Eotetranychus* Oudemans (Acariens: Tetranychidae) de Madagascar	Acarologia, 9: 370–394	yes	32
*Eotetranychus*	*savanae*	Gutierrez, 1967	*Eotetranychus savanae*	Cinq autres nouvelles espèces de Tetranychidae de Madagascar (Troisième note)	Acarologia, 9: 567–580	yes	33
*Eotetranychus*	*tulearensis*	Gutierrez, 1967	*Eotetranychus tulearensis*	Huit nouvelles espèces du genre *Eotetranychus* Oudemans (Acariens: Tetranychidae) de Madagascar	Acarologia, 9: 370–394	yes	36
*Eotetranychus*	*xylopiae*	Gutierrez, 1970	*Eotetranychus xylopiae*	Tetranychidae nouveaux de Madagascar (Sixième note)	Acarologia, 12: 714–731	yes	18
*Eurytetranychus*	*madagascariensis*	Gutierrez, 1966	*Eurytetranychus madagascariensis*	Cinq nouvelles espèces de Tetranychidae de Madagascar	Acarologia, 8: 594–610	yes	7
*Eutetranychus*	*eliei*	Gutierrez & Helle, 1971	*Eutetranychus eliei*	Deux nouvelles espèces du genre *Eutetranychus* Banks (Acariens: Tetranychidae) vivant sur plantes cultivées à Madagascar	Entomologische Berichten, Amsertdam, 31: 45–60	yes	16
*Eutetranychus*	*grandidieri*	Gutierrez, 1966	*Aponychus grandidieri*	Cinq nouvelles espèces de Tetranychidae de Madagascar	Acarologia, 8: 594–610	yes	19
*Eutetranychus*	*ranjatoi*	Gutierrez, 1967	*Duplanychus ranjatoi*	Cinq autres nouvelles espèces de Tetranychidae de Madagascar (Troisième note)	Acarologia, 9: 567–580	yes	31
*Hellenychus*	*bollandi*	Gutierrez, 1970	*Hellenychus bollandi*	Tetranychidae nouveaux de Madagascar (Sixième note)	Acarologia, 12: 714–731	yes	27
*Oligonychus*	*andrei*	Gutierrez, 1966	*Oligonychus andrei*	Cinq nouvelles espèces de Tetranychidae de Madagascar	Acarologia, 8: 594–610	yes	34
*Oligonychus*	*andropogonearum*	Gutierrez, 1969	*Oligonychus andropogonearum*	Tetranychidae nouveaux de Madagascar (Cinquième note)	Acarologia, 11: 43–64	yes	6
*Oligonychus*	*bessardi*	Gutierrez, 1966	*Oligonychus bessardi*	Cinq nouvelles espèces de Tetranychidae de Madagascar	Acarologia, 8: 594–610	yes	26
*Oligonychus*	*chazeaui*	Gutierrez, 1970	*Oligonychus chazeaui*	Tetranychidae nouveaux de Madagascar (Sixième note)	Acarologia, 12: 714–731	yes	22
*Oligonychus*	*etiennei*	Gutierrez, 1982	*Oligonychus etiennei*	Deux acariens phytophages vivant sur canne a sucre a la Réunion: *Oligonychus etiennei* n.sp. (Tetranychidae) et *Abacarus sacchari* (Eriophyidae)	Agronomie Tropicale, 37: 389–392	yes	22
*Oligonychus*	*hova*	Gutierrez, 1966	*Oligonychus hova*	Cinq nouvelles espèces de Tetranychidae de Madagascar	Acarologia, 8: 594–610	yes	31
*Oligonychus*	*leandrianae*	Gutierrez, 1970	*Oligonychus leandrianae*	Tetranychidae nouveaux de Madagascar (Sixième note)	Acarologia, 12: 714–731	yes	2
*Oligonychus*	*monsarrati*	Gutierrez, 1967	*Oligonychus monsarrati*	Cinq autres nouvelles espèces de Tetranychidae de Madagascar (Troisième note)	Acarologia, 9: 567–580	yes	45
*Oligonychus*	*occidentalis*	Gutierrez, 1969	*Oligonychus occidentalis*	Tetranychidae nouveaux de Madagascar (Cinquième note)	Acarologia, 11: 43–64	yes	14
*Oligonychus*	*pemphisi*	Gutierrez, 1970	*Oligonychus pemphisi*	Tetranychidae nouveaux de Madagascar (Sixième note)	Acarologia, 12: 714–731	yes	15
*Oligonychus*	*randriamasii*	Gutierrez, 1967	*Oligonychus randriamasii*	Cinq autres nouvelles espèces de Tetranychidae de Madagascar (Troisième note)	Acarologia, 9: 567–580	yes	54
*Oligonychus*	*senegalensis*	Gutierrez & Etienne, 1981	*Oligonychus senegalensis*	Une nouvelle espèce du genre *Oligonychus* (Acariens: Tetranychidae) attaquant le riz au Sénégal	Agronomie Tropicale, 36: 389–390	yes	13
*Oligonychus*	*thelytokus*	Gutierrez, 1977	*Oligonychus thelytokus*	Un tétranyque polyphage de la zone intertropicale: *Oligonychus thelytokus* sp. n.	Cahiers de l’ORSTOM, série Biologie, 12: 65–72	yes	9
*Oligonychus*	*tiwakae*	Gutierrez, 1978	*Oligonychus tiwakae*	Cinq nouvelles espèces de Tetranychidae (Acariens) de Nouvelle-Calédonie	Acarologia, 20: 351–364	yes	15
*Oligonychus*	*virens*	Gutierrez, 1969	*Oligonychus virens*	Tetranychidae nouveaux de Madagascar (Cinquième note)	Acarologia, 11: 43–64	yes	5
*Schizonobia*	*bundi*	Gutierrez, 1972	*Schizonobia bundi*	Récolte, dans le Var, d’une espèce appartenant à un genre nouveau pour la France: *Schizonobia bundi* sp. n. (Acariens: Tetranychidae)	Acarologia, 14: 379–383	no	0
*Schizonobia*	*oudemansi*	Gutierrez & Bolland, 1986	*Schizonobia oudemansi*	Description and karyotype of *Schizonobia oudemansi* sp. n. from The Netherlands (Acari: Tetranychidae)	Entomologische Berichten, Amsertdam, 46: 39–43	yes	10
*Schizotetranychus*	*australis*	Gutierrez, 1968	*Schizotetranychus australis*	Tetranychidae nouveaux de Madagascar (Quatrième note)	Acarologia, 10: 13–28	yes	38
*Schizotetranychus*	*fauveli*	Gutierrez, 1978	*Schizotetranychus fauveli*	Cinq nouvelles espèces de Tetranychidae (Acariens) de Nouvelle-Calédonie	Acarologia, 20: 351–364	yes	19
*Schizotetranychus*	*tephrosiae*	Gutierrez, 1968	*Schizotetranychus tephrosiae*	Tetranychidae nouveaux de Madagascar (Quatrième note)	Acarologia, 10: 13–28	yes	22
*Tetranychus*	*kaliphorae*	Gutierrez, 1969	*Tetranychus kaliphorae*	Tetranychidae nouveaux de Madagascar (Cinquième note)	Acarologia, 11: 43–64	yes	25
*Tetranychus*	*montrouzieri*	Gutierrez, 1978	*Tetranychus montrouzieri*	Cinq nouvelles espèces de Tetranychidae (Acariens) de Nouvelle-Calédonie	Acarologia, 20: 351–364	yes	6
*Tetranychus*	*panici*	Gutierrez, 1969	*Tetranychus panici*	Tetranychidae nouveaux de Madagascar (Cinquième note)	Acarologia, 11: 43–64	yes	21
*Tetranychus*	*roseus*	Gutierrez, 1969	*Tetranychus roseus*	Tetranychidae nouveaux de Madagascar (Cinquième note)	Acarologia, 11: 43–64	yes	41
*Tetranychus*	*tchadi*	Gutierrez & Bolland, 1973	*Tetranychus tchadi*	Description et caryotype d’une nouvelle espèce du genre *Tetranychus* Dufour (Acariens:Tetranychidae) récoltée au Tchad sur *Dolichos lablab* L. (Papilionaceae)	Entomologische Berichten, Amsertdam, 33: 155–158	yes	33
*Trichonychus*	*insularis*	Gutierrez, 1968	*Porcupinychus insularis*	Tetranychidae nouveaux de Madagascar (Quatrième note)	Acarologia, 10: 13–28	yes	43

## Taxonomic ranks

**Kingdom:** Animalia.

**Phylum:** Arthropoda.

**Class:** Arachnida.

**Order:** Trombidiformes.

**Family:** Tetranychidae.

### Spatial coverage

**Figure 1.**
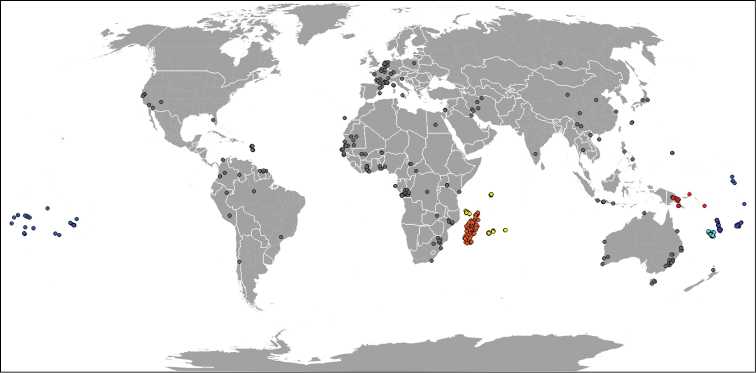
World map representing all the locations mentioned in the dataset. Areas of particular interest are represented with the same colour (⬤ Madagascar, ⬤ Western Indian Ocean, ⬤ Papuasia, ⬤ New Caledonia, ⬤ South Pacific). Grey spots gather all the other locations.

**Figure 2.**
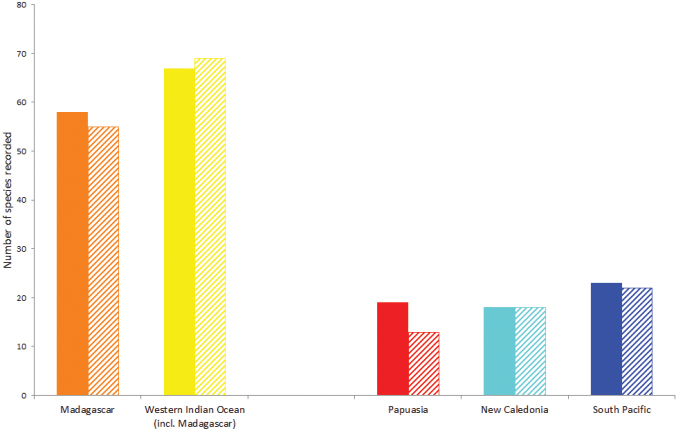
Number of species recorded in Jean Gutierrez collection dataset (solid bar) and in the literature (dashed bar) compiled in Spider Mites Web (http://www1.montpellier.inra.fr/CBGP/spmweb/) for the areas of particular interest. Colour scheme same as in Figure 1.

The spatial coverage varies among geographic areas (Figure 1) most being collected in Madagascar and Western Indian Ocean or in New Caledonia, South Pacific and Papuasia. Not all specimens from these areas were mentioned in the literature (Figure 2) compiled in Spider Mites Web (Migeon and Dorkeld 2006–2013).

### Temporal coverage

1963–1999.

### Natural collections description

**Collection name:**

Spider Mites collection of Jean Gutierrez.

**Specimen preservation methods:**

Specimens are preserved on microslides mounted with Hoyer medium after clearing in lactic acid and coloring with lignin pink (Gutierrez 1985). Microslides boxes are stored in the CBGP collection room maintained at 20 +/- 2 °C and 25 +/- 10% RH.

## Methods

### Method step description

There are 5,262 microscopic slides recorded in the dataset. Each one contains a single specimen. Specimens identified at genus level only, without location data, or from laboratory breeding have been discarded, for a total of 347 specimens. All (and only) indications given on the label have been recorded. Location coordinates (Decimal degrees – DD – WGS84 geodetic system) have been assigned using several geolocation tools like GoogleMaps, GeoNames and other gazetteers, completed when necessary by textual search. Country and TDWG level 4 polygon were assigned to each location (http://www.tdwg.org/standards/109/)

### Uncertainty issues

Unknown collection date was set as 1^st^ January 1901 for 31 specimens. This convention takes advantage to be outside of the temporal range of Jean Gutierrez work, indicating the absence of temporal data. When only year was reported date was set as 1^st^ January of the year. When only month and year were reported, date was set as 15^th^ of the month.

Location precision has been assigned from 0.01° DD when the place was found corresponding to a small area (1–10 km²), 0.1° DD when place was corresponding to a bigger area (10–100 km²), 0.5° DD (100–2500 km²), to 1° DD (2500–10000 km²). For one slide it was not possible to assign coordinates (location not found). Then only country reported on the label has been published.

### Quality control description

The Tetranychidae nomenclature is in accordance with current reference: Spider Mites Web (Migeon and Dorkeld 2006–2013) and Catalogue of Life (Roskov et al. 2014). Determinations have been performed by Jean Gutierrez himself a well-known and internationally recognized specialist (Bolland et al. 1998). In case of doubt, identification was checked and rectified before publication with present knowledge if necessary. Host plant nomenclature is in accordance to current reference (The Plant List 2013). Geographic coordinates were visually verified using the Check Coordinates tool in Diva-GIS (Hijmans et al. 2012) and manual verification (points in the sea…).

## Dataset

Object name: Darwin Core Archive Spider Mites collection of Jean Gutierrez.

Character encoding: UTF-8.

Format name: Darwin Core Archive Format.

Format version: 1.0.

Distribution: http://www.gbif.org/dataset/ac60a288-fcc9-43fe-a7d4-e732b748a981

Publication date of data: 2014-06-18

Language: English

License of use: Open Data Commons Attribution License (ODC-By).

## References

[B1] Blommers L Gutierrez J (1975) Les tétranyques vivant sur agrumes et avocatiers dans la région de Tamatave (Madagascar-est) et quelques-uns de leurs prédateurs. Fruits 30: 191–200.

[B2] Bolland HR Gutierrez J Flechtmann CHW (1998) World catalogue of the spider mite family (Acari: Tetranychidae). Brill Academic Publishers, Leiden, 392 pp.

[B3] Gutierrez J (1966) Cinq nouvelles espèces de Tetranychidae de Madagascar. Acarologia 8: 594–610.

[B4] Gutierrez J (1967a) Cinq autres nouvelles espèces de Tetranychidae de Madagascar (Troisième note). Acarologia 9: 567–580.

[B5] Gutierrez J (1967b) Huit nouvelles espèces du genre *Eotetranychus* Oudemans (Acariens: Tetranychidae) de Madagascar. Acarologia 9: 370–394.

[B6] Gutierrez J (1968a) Note sur quelques acariens phytophages de l’Ile de la Réunion avec description d’une nouvelle espèce du genre *Eotetranychus* Oudemans (Tetranychidae). Acarologia 10: 444–446.

[B7] Gutierrez J (1968b) Tetranychidae nouveaux de Madagascar (Quatrième note). Acarologia 10: 13–28. 5806300

[B8] Gutierrez J (1969) Tetranychidae nouveaux de Madagascar (Cinquième note). Acarologia 11: 43–64. 5806300

[B9] Gutierrez J (1970) Tetranychidae nouveaux de Madagascar (Sixième note). Acarologia 12: 714–731. 5806300

[B10] Gutierrez J (1972) Récolte, dans le Var, d’une espèce appartenant à un genre nouveau pour la France: *Schizonobia bundi* sp. n. (Acariens: Tetranychidae). Acarologia 14: 379–383.

[B11] Gutierrez J (1977) Un tétranyque polyphage de la zone intertropicale: *Oligonychus thelytokus* sp. n. Cahiers de l’ORSTOM, série Biologie 12: 65–72.

[B12] Gutierrez J (1978) Cinq nouvelles espèces de Tetranychidae (Acariens) de Nouvelle-Calédonie. Acarologia 20: 351–364.

[B13] Gutierrez J (1982) Deux acariens phytophages vivant sur canne à sucre à la Réunion: *Oligonychus etiennei* n.sp. (Tetranychidae) et *Abacarus sacchari* (Eriophyidae). Agronomie Tropicale 37: 389–392.

[B14] Gutierrez J Bolland HR (1973) Description et caryotype d’une nouvelle espèce du genre *Tetranychus* Dufour (Acariens:Tetranychidae) récoltée au Tchad sur *Dolichos lablab* L. (Papilionaceae). Entomologische Berichten, Amsertdam 33: 155–158.

[B15] Gutierrez J Bolland HR (1986) Description and karyotype of *Schizonobia oudemansi* sp. n. from The Netherlands (Acari: Tetranychidae). Entomologische Berichten, Amsertdam 46: 39–43.

[B16] Gutierrez J Etienne J (1981) Une nouvelle espèce du genre *Oligonychus* (Acariens: Tetranychidae) attaquant le riz au Sénégal. Agronomie Tropicale 36: 389–390.

[B17] Gutierrez J Helle W (1971) Deux nouvelles espèces du genre *Eutetranychus* Banks (Acariens: Tetranychidae) vivant sur plantes cultivées à Madagascar. Entomologische Berichten, Amsertdam 31: 45–60.

[B18] Hijmans RJ Guarino L Bussink C Marthur P Cruz M Barrentes I Rojas E (2012) DIVA-GIS. 7.5. A geographic information system for the analysis of species distribution data. Manual available at http://www.diva-gis.org

[B19] Migeon A Dorkeld F (2006-2013) Spider Mites Web: a comprehensive database for the Tetranychidae. http://www.montpellier.inra.fr/CBGP/spmweb [accessed 2014-06-27]

[B20] Roskov Y Abucay L Orrell T Nicolson D Kunze T Culham A Bailly N Kirk P Bourgoin T DeWalt RE Decock W De Wever A (2014) Species 2000 & ITIS Catalogue of Life, 22nd December 2014 available at http://www.catalogueoflife.org/col [accessed 2015-01-26]. In: Roskov Y Abucay L Orrell T Nicolson D Kunze T Culham A Bailly N Kirk P Bourgoin T DeWalt RE Decock W De Wever A (Eds) Species 2000: Naturalis, Leiden, the Netherlands.

[B21] The Plant List Version 1.1. http://www.theplantlist.org [accessed 2014-06-27]

